# Ocular extraintestinal manifestations and treatments in patients with inflammatory bowel disease

**DOI:** 10.3389/fopht.2023.1257068

**Published:** 2024-01-04

**Authors:** Mariana Rodriguez Duran, Ghazala A. Datoo O’Keefe

**Affiliations:** Department of Ophthalmology, Emory University School of Medicine, Atlanta, GA, United States

**Keywords:** uveitis, Inflammatory bowel disease, episcleritis, Crohn’s disease, ulcerative colitis, scleritis, retinal vasculitis, treatment

## Abstract

Between 3-47% of patients with inflammatory bowel disease (IBD) have extraintestinal manifestations (EIMs), and between 1.3-86.9% of patients with IBD suffer from ocular EIMs (O-EIMs) making the eye the third most common organ affected. These O-EIMs exist among a spectrum, with a variety of types and amounts of inflammation which can lead to decreased vision, and in some cases, vision loss, without treatment. We performed a literature review concerning O-EIMs in patients who had or were later found to have a diagnosis of IBD in order to identify ocular EIMs that commonly occur with IBD and to assess which patients with IBD may be at higher risk of developing O-EIMs. We were also interested in ascertaining whether O-EIMs were more common in specific populations of people or in specific subtypes of IBD. Lastly, we explored the common treatments of O-EIMs in patients with IBD. Upon review of the literature, we found that the most common O-EIMs are episcleritis and uveitis. Anterior uveitis is more commonly seen, although, inflammation may occur in the posterior segment of the eye as well and may also manifest as retinal vasculitis. While these diagnoses are sometimes known retrospectively, most patients present with nonspecific eye complaints of which decreased vision with or without pain is the most common. Visual symptoms associated with ocular EIMs may be non-specific so physicians should have a low threshold to refer to ophthalmology for visual complaints. It is important to keep in mind that ocular EIMs can cluster with skin and joint EIMs. Screening should be prioritized for female patients with Crohn’s disease and concurrent arthritis. Treatments for O-EIMs are outlined and compared in this paper as well.

## 1 Introduction

Inflammatory bowel disease (IBD) is a chronic, autoimmune condition that causes inflammation in the gastrointestinal tract (1). It can occur in adults and in children and is comprised of two separate diagnoses: ulcerative colitis (UC) and Crohn’s disease (CD). While UC is a contiguous process that involves the mucosal lining of the large intestine, CD presents as skip lesions that can occur anywhere along the digestive tract and can involve the whole thickness of the wall of the digestive tract. Nonetheless, both UC and CD present as recurrent, acute gastrointestinal attacks (1). These recurrent attacks cause a great impact on the patient’s quality of life and can disrupt career and personal aspirations (2). The majority of diagnoses are made when patients are in adolescence or in early adulthood (3).

IBD has been believed to be a disease of industrialized nations with initial rates of disease being higher in Western countries and increasing rates in countries as they become more industrialized (3–5). While appendectomy and cigarette smoking are the strongest environmental factors identified linking IBD with industrialized nations, it is still unclear whether other factors including diet, infections, and oral contraceptive use plays a role in the development of IBD (3). Currently, there is a global increase in the burden of inflammatory bowel disease which is associated with increased healthcare costs worldwide (3).

In addition to gastrointestinal symptoms, patients with IBD present with extra-intestinal manifestations (EIMs) of the disease. These manifestations present in, but are not limited to, the skin, joints, and the eyes. The prevalence of EIMs ranges from 3% (6) to 47% (7–12) in patients with inflammatory bowel disease (IBD). Among those with EIMs, ocular EIMs (O-EIMs) are seen between 1.3% (13) to 86.9% (14) of patients, following around 20% with skin involvement (15) and up to 33% with joint involvement (16). The eye is the third most common organ affected. Manifestations in the eye exist along a spectrum, with a variety of presentations and varying amounts of inflammation which can lead to decreased vision, and in some cases, vision loss if treatment is not initiated in a timely fashion.

We performed a literature review evaluating the concurrence of ocular manifestations of IBD and the diagnosis of IBD in patients. We performed the literature review in PubMed and Google Scholar from 1967 to 2022 of articles written in English or translated to English, and we used a combination of the words “uveitis”, “inflammatory bowel disease”, “Crohn’s disease”, “ulcerative colitis”, “treatment”, “episcleritis”, “scleritis”, “retinal vasculitis” to refine our search. We found 406 articles, many of which were case reports; however, we only included published peer-reviewed articles that provided data on the incidence, prevalence, clinical features, or management of ophthalmic manifestations in patients with IBD. We reviewed the rates at which diseases occur and risk factors that may be associated with an increased rate of developing O-EIMs in patients. The sample sizes, demographics, and prevalence of (O-)EIMs of the studies included can be found in **Table 1**.

**Table 1:** Sample Sizes, Demographics and Prevalences of Studies Included.

Reference	Article Type	Years	Location	Sample Size	Prevalence of (O)-EIMs
Barberio et al. (17)	Observational Cohort	2020	United States of America	179,222,705	Not reported
Card et al. (18)	Retrospective Chart Review	2016	United Kingdom	56,097	Uveitis: 3.4%
Dubinsky et al. (19)	Retrospective Cohort	2012-2016	United States of America	18,055	Not reported
Charlton et al. (20)	Population Cohort	1998-2014	United Kingdom	6,783	Uveitis: 1.55-9.09%
Bernstein et al. (9)	Population Cohort	2001	Canada	4,454	EIMs: 6.2%
Biedermann et al. (21)	Population Cohort	2019	Switzerland	3,266	Not reported
Barisani-Asenbauer et al. (22)	Review	1995-2009	Austria	2,619	Uveitis: 38 cases per 100,000 people
Karmiris et al. (23)	Population Cohort	2016	Greece	1,860	EIMs: 16-40%
Jose et al. (6)	Cohort	2009	United States of America	1,649	EIMs: 3-7%
Castro et al. (24)	Population Cohort	1996-2003	Italy	1,576	Not reported
Orchard et al. (11)	Retrospective Chart Review	1998	United Kingdom	1,459	Not reported
Vavricka et al. (7, 8)	Population Cohort	2015	Switzerland	1,249	EIMs: 6-47%
Rubin et al. (25)	Randomized Control Trial	2012-2016	United States of America	1,139	Not reported
Dotson et al. (26)	Prospective Observational	2010	United States of America	1,009	Not reported
Vavricka et al. (27)	Prospective Cohort	2011	Switzerland	950	EIMs: 31-43% O-EIMs: 10%
Lakatos et al. (28)	Observational Cohort	2003	Hungary	873	EIMs: 21.3%
Truyens et al. (13)	Retrospective Cohort	2021	Belgium, Spain	856	EIMs: 20.9% O-EIMs: 1.3%
Veloso et al. (29)	Prospective Cohort	1996	Portugal	792	EIMs: 25.8% O-EIMs: 4.6-6.4%
Palm et al. (30)	Population Cohort	1997-2002	Norway	654	Not reported
Rankin et al. (12)	Case Series	1979	United States of America	569	EIMs: 24%
Mendoza et al. (11)	Prospective Cross-Sectional	2005	Spain	566	EIMs: 46.6%
Dimakou et al. (31)	Retrospective Chart Review	1981-2011	Greece	483	Not reported
Feagan et al. (32)	Randomized Control Trial	2016	United Kingdom, Canada, United States of America, Belgium, South Africa, Hungary, Germany, France	397	Not reported
Gangaputra et al. (33)	Retrospective Cohort	1979-2007	United States of America	384	Not reported
Suhler et al. (34)	Randomized Control Trial	2018	United States of America, Spain, France, Japan, Canada, Israel, Australia, Brazil, Belgium, Italy, Germany, Argentina, Netherlands, United Kingdom	371	Not reported
Cloche et al. (35)	Cross-Sectional	2009-2011	France	305	O-EIMs: 32%
Ricart et al. (10)	Case-Control	2004	United States of America	243	EIMs: 40%
Nguyen at al (36).	Randomized Control Trial	2016	United States of America, Canada, Israel, Australia, United Kingdom, Argentina, Italy, Belgium, Germany, France	226	Not reported
Jaffe et al. (37)	Randomized Control Trial	2016	United States of America, United Kingdom, France, Belgium, Austria, Argentina, Germany	217	Not reported
Hofley et al. (38)	Cross-Sectional Prospective	1993	Canada	147	O-EIMs: 6.2%
Cakir et al. (39)	Retrospective Chart Review	2004-2012	Turkey	127	EIMs: 20%
Yilmaz et al. (40)	Randomized Control Trial	2007	Turkey	116	Not reported
Yilmaz et al. (40)	Randomized Control Trial	2007	Turkey	116	Not reported
Cury et al. (41)	Prospective Cohort	2010	Brazil	112	O-EIMs: 42%
Kopylov, U. et al. (42)	Retrospective Cohort	2021	Belgium, Denmark, Israel, Netherlands and Switzerland	99	Uveitis: 1%
Funk et al. (43)	Prospective Cross-Sectional	2021	United States of America	97	Not reported
Rohani et al. (44)	Case Series	2014-2019	Iran	96	O-EIMs: 6.25%
Naviglio et al. (45)	Observational Cohort	2017	Italy	94	O-EIMs: 2.13%
Walldorf et al. (14)	Prospective Cohort	2018	Germany	61	O-EIMs: 86.9%
Felekis et al. (46)	Prospective Cohort	2009	Greece	60	O-EIMs: 43%
Gui et al. (47)	Clinical Trial	1997	United Kingdom	52	Not reported
Martel et al. (48)	Retrospective Longitudinal Case Series	2012	United States of America	43	Not reported
Shafran et al. (49)	Open Clinical Trial	2002	United States of America	36	Not reported
Rychwalsky et al. (50)	Prospective Cohort	1994-1995	United States of America	32	Uveitis: 12.5%
Suhler et al. (51)	Randomized Control Trial	2009	United States of America	32	Not reported
Daum et al. (52)	Case Series	1967	United States of America	19	Uveitis: 31.6%
Lyons et al. (53)	Case-Control	1997	United States of America	17	O-EIMs: 59%
Korelitz et al. (54)	Case Series	2022	United States of America	13	Not reported
Chateau et al. (55)	Case Report	2019	France	2	Not reported
Baiocco et al. (56)	Case Report	1984	United States of America	1	Not reported
Salmon et al. (57)	Case Report	1989	South Africa	1	Not reported
Sykes et al. (58)	Case Report	1997	United States of America	1	Not reported
Saatci et al. (59)	Case Report	2001	Turkey	1	Not reported
Freeman (60)	Case Report	2001	Canada	1	Not reported
Fries et al. (61)	Case Report	2002	Italy	1	Not reported
Awotesu et al. (62)	Case Report	2004	United Kingdom	1	Not reported
Singleton et al. (63)	Case Report	2006	United States of America	1	Not reported
Girardin et al. (64)	Case Report	2007	Canada	1	Not reported
Haider et al. (65)	Case Report	2007	United Kingdom	1	Not reported
Wang, F & Wang, NS (66)	Case Report	2009	China	1	Not reported
Paroli et al. (67)	Case Report	2011	Italy	1	Not reported
Figueiredo et al. (68)	Case Report	2014	Portugal	1	Not reported
Singla et al. (69)	Case Report	2015	United States of America	1	Not reported
Shapiro et al. (70)	Case Report	2016	United States of America	1	Not reported
Bancu et al. (71)	Case Report	2016	Romania	1	Not reported
Coussa et al. (72)	Case Report	2016	Canada	1	Not reported
Chandra et al. (73)	Case Report	2021	India	1	Not reported
Kilgore et al. (74–76)	Case Report	2022	United States of America	1	Not reported

## 2 Presence of O-EIMs in IBD

In adults, ocular manifestations of IBD vary broadly from surface pathologies including dry eye to blinding eye disease, such as scleritis and uveitis. Prevalence rates vary broadly in the published literature. Dry eye has a prevalence of up to 44% across multiple populations (30, 41, 46). Episcleritis has a prevalence of 4% as compared with scleritis which has a prevalence of <1% (7–9, 14, 21, 23, 35, 40, 77). Anterior uveitis has a prevalence of 5%, and other locations of uveitis have a prevalence of <1% (14, 21, 35). Of these, some studies delineate the specific form of uveitis, while others group them as one diagnosis. Most studies cite a prevalence rate of 6.2% (38, 44) of ocular EIMs where dry eye is commonly excluded.

Fewer studies have been performed in the pediatric population to assess the prevalence of O-EIMs in children with IBD (38, 45, 78, 79). A study from Iran found that 6 of 96 children (6.2%) with IBD had ocular manifestations (26); though other studies cite a lower prevalence of 0.7% to 1.8% (6, 39, 45, 79). One study offered ophthalmic exams to pediatric patients with IBD in a gastroenterology clinic in Italy and found one patient out of 94 participants with asymptomatic uveitis without intraocular complications (45). The burden of ocular EIMs in children with IBD appears to be lower than in adults, but not insignificant. Further studies should be performed in the pediatric population to better assess the prevalence of O-EIMs and the disease course to identify the risk of vision loss.

Adult patients with Crohn’s Disease (CD) are more likely to have ocular manifestations of their inflammatory disease than patients with Ulcerative Colitis (UC) (21, 28, 38, 40). A similar trend exists in the pediatric population despite the limited studies in the pediatric population (78). Larger pediatric studies have demonstrated that patients with CD are more likely to have anterior uveitis and episcleritis (18, 24, 26, 31, 39). The predominance of O-EIMs in patients with CD rather than UC in adult and pediatric patients can be due to the fact that CD has a greater surface area of inflammation in the gastrointestinal system indicating a more systemic inflammation which can involve the eyes (1).

In line with trends of autoimmune diseases (80), women seem to develop O-EIMs more frequently than men (28, 30, 81). The prevalence of ocular EIMs in women is 2.2-6.7% compared with 1.1-4.4% in men (30, 39). In terms of prevalence of O-EIMs in patients with UC verses CD in females, a large Swiss cohort study found more O-EIMs in females with CD, but not in those with UC (39), while a population study from the University of Manitoba found O-EIMs to be the most common in women with UC (30). Numerous other studies (28, 30, 81) have not been able to identify a specific gender correlation with a sub-type (28, 30, 80, 81). Therefore, it has been understood that female sex and CD are independent predictors for the development of O-EIMs (27).

## 3 Ocular presentations

### 3.1 Initial flare

The onset of O-EIM symptoms vary broadly in relation to IBD symptoms. Patients with episcleritis tend to flare with bowel disease activity (53). On the other hand, patients with uveitis appear to flare independently of bowel disease activity (53). In pediatric patients, a cross-sectional prospective study found that there is no relationship between bowel disease activity and ocular inflammation (38). However, this data is limited.

Patients may present to an ophthalmologist with findings of eye inflammation prior to receiving a diagnosis of gut inflammation. A case report identified a patient with an initial presentation of posterior uveitis that went on to develop symptoms of IBD 6 months after the onset of uveitis (74). This is not the only case of ophthalmic presentation preceding gastrointestinal symptoms (70). Another case report described a patient who developed anterior uveitis eight years prior to developing bowel inflammation (67). For these patients who present initially with isolated uveitis, the differential diagnosis can be quite broad and an ongoing review of symptoms can help identify a new inflammatory disease that manifests over the course of treatment.

Patients with known IBD are referred to ophthalmology upon developing ocular symptoms (64). The vast majority will have symptoms of dry eyes, but a significant minority will go on to develop scleritis or uveitis. A study that performed ophthalmic screenings in a gastroenterology clinic in patients with IBD demonstrated occult eye disease which was defined as asymptomatic anterior chamber cell or flare (50, 67).

### 3.2 Risk of developing ocular manifestations

Unsurprisingly, HLA genes have also been found to be biomarkers of EIMs of IBD, and while some genetic associations, such as B27, B58, and DR 103, have been implicated, the phenotypic manifestations can vary broadly (63, 81). One study speculates that the disequilibrium between the HLA genes may be the culprit of EIMs (81); however, no studies have been done to date to assess this theory.

Various studies have proposed that patients having one EIM are at higher risk of developing another EIM (29, 71, 82). A prospective study looking at EIMs found a strong association of arthritis with having eye, skin, and mouth complications of IBD (29). A large cohort study evaluating the risk of uveitis in IBD patients with psoriatic arthritis found an increased risk of 3.55 of developing uveitis in patients with CD (20). Similarly, a cross-sectional study assessing patients with IBD in the ophthalmology clinic found arthritis was highly prevalent among the uveitis patients indicating a strong association between uveitis and arthritis EIMs (22). Another prospective study found that having uveitis was directly associated with the onset of arthritis and erythema nodosum in patients with IBD (41). They also proposed that the presence of uveitis may be used to predict disease severity. This finding has been replicated and research suggests that the gene encoding the RBM19 locus may serve as a biomarker for the concurrence of O-EIMs (83).

## 4 Manifestations

Patients with IBD who develop O-EIMs most commonly complain of dry eyes (40, 41, 82). While there exists a spectrum of disease ranging from eye pain secondary to dry eye (69, 74) to uveitis causing vision loss and enucleation (57) from complications, most eye complaints are non-specific. Ocular manifestations in children may be hard to detect, with one case report discussing the presence of disc edema and choroidal thickening as the only finding on examination in a child with IBD who had failed a school screening exam (74). Other case reports of children with IBD have described anterior and intermediate uveitis in addition to posterior segment findings (74–76).

The most common O-EIMs are episcleritis and anterior uveitis, where the prevalence of anterior uveitis (0.7-11%) is more common than other types of uveitis (<1%), including posterior uveitis (7–9, 14, 21, 23, 35, 40, 74, 77). A retrospective study from 1967 found that 1.9% of patients with uveitis associated with IBD almost always involved the anterior uvea (54). Studies which performed full ophthalmic screenings of patients with IBD of whom the majority had CD, most commonly found mild anterior uveitis (38, 50, 52). Patients have also been reported to present with retinal vasculitis despite remission of their gut disease. The retinal vasculitis is usually steroid responsive, though patients may present with fulminant retinal vasculitis, with vascular occlusion due to vasculitis, or with neovascularization that needs to be treated urgently to prevent vision loss (58, 59, 68, 72, 73, 84).

## 5 Treatments

For patients who present with anterior uveitis, episcleritis, or scleritis, topical corticosteroid drops or oral non-steroidal anti-inflammatory drugs can be a temporary treatment. However, care must be taken due to the presence of inflammatory bowel disease and the relative contraindication of oral NSAIDs (78).

Mild cases of uveitis can be treated with topical corticosteroids; however, it is common to progress to a systemic corticosteroid use if there is no improvement or in severe or recalcitrant cases (16, 40, 78, 85). Systemic steroids have been commonly used to effectively treat autoimmune diseases including IBD for many years (56, 60, 74). However, they cause a plethora of undesirable systemic side effects as well as increased intraocular pressure and cataract formation which can be prevented by utilizing corticosteroid-sparing agents (78).

### 5.1 Anti-metabolites

Anti-metabolites, namely methotrexate, azathioprine, and mycophenolate mofetil, prevent DNA synthesis and are frequently used as first line alternatives to corticosteroids. Methotrexate, which prevents DNA synthesis through inhibiting dihydrofolate reductase, has demonstrated effective results in the treatment of ocular inflammation as well as other autoimmune conditions. A multicenter, retrospective cohort study evaluating methotrexate’s treatment outcomes in patients with non-infectious ocular inflammation found that methotrexate is moderately effective for achieving a corticosteroid-sparing drug regimen (33). However, for patients with active luminal CD, methotrexate is more effective with a tapering dose of corticosteroids (86). Methotrexate is used more frequently than azathioprine and mycophenolate mofetil due to its once weekly dosing, and long term availability of safety data (86). However, in the settings of pregnancy or lactation, azathioprine is preferred (86). These medications are more inexpensive than the newer immune modulator treatments and have fewer systemic side effects than the corticosteroid medications; however, their broad mechanism of action still predisposes patients to a variety of other side effects. Newer agents such as biologic response modifiers have been noted to have fewer side-effects.

### 5.2 Anti-TNF alpha medications

Anti-tumor necrosis factor (TNF) alpha agents comprise the main class of drugs referred to as biologics. The main stay of therapy for inflammatory bowel disease utilizes biologic therapies (86). TNF-alpha antagonist medications are monoclonal antibodies that bind to the TNF-alpha antigen to prevent pro-inflammatory effects (87). Infliximab (Remicade), adalimumab (Humira), and etanercept (Enbrel) comprise the most common TNF-alpha antagonists. These medications have been used to treat patients with IBD along with other non-ophthalmic autoimmune conditions and have been widely used off-label for the treatment of refractory uveitis.

Infliximab has been found to not only treat the underlying IBD symptoms, but also the EIMs (86). A cross-sectional and prospective study found that higher doses are required to effectively treat EIMs when compared to maintenance dosing for IBD symptoms due to an elevated inflammatory burden as measured by infliximab clearance and serum anti-drug metabolites (43). It is important to note, however, that the study (43) was done in children of whom only 8 had uveitis.

With a relatively low rate of treatment-ending adverse events at about 19%, infliximab has been shown to be a safe medication for patients who meet criteria (48, 51). There has been a case report, however, of a patient with UC who developed anterior uveitis after a second infusion of infliximab (69). Though unusual, the timing of the infusion and resolution with discontinuation point to infliximab as the likely cause, though this is a rare finding, and infliximab has demonstrated success in achieving remission from refractory uveitis with eventual and successful taper off the drug (51). A large observational cohort study found no difference in the risk of developing uveitis in patients who were taking infliximab versus adalimumab or an antimetabolite (azathioprine or methotrexate) thus indicating that antimetabolites and TNF-inhibitors are equally effective at suppressing uveitis (17). For patients who meet criteria for TNF-alpha antagonists and corticosteroid-sparing antimetabolites, the antimetabolites offer a more cost-effective option though they come with greater side-effects.

Adalimumab was shown to be effective at treating non-infectious uveitis (NIU) in VISUAL I and II studies which were multicenter randomized, controlled trials. In these trials (36, 37), the authors found a low risk of treatment failure in patients being treated with adalimumab versus placebo and the risk of adverse events was similar between the two groups as well. In the VISUAL III study (34), they found improved control of ocular inflammation, improvement, and stabilization of BCVA, and reduced corticosteroid usage. Additionally, a study analyzing 75 clinical trials of adalimumab in patients with varying inflammatory diseases found no incidence of IBD in patients with uveitis (88). Adalimumab has been widely used for IBD management, and with its effectiveness in treating uveitis as well, it is a common choice for patients who may benefit from a biologic response modifier.

Etanercept should be used cautiously in patients with ankylosing spondylitis since there have been case reports of new onset uveitis (66) and CD (19) in patients while on etanercept. However, no cases have been reported of uveitis development in a patient with IBD or vice versa while taking etanercept.

### 5.3 Integrins

Vedolizumab is a gut-selective alpha4beta7 integrin blocker that has been approved for moderately to severely active UC and CD in adults. A multicenter retrospective study found that patients with EIMs had 50% improvement at 12 months. The study had 1 patient with uveitis who demonstrated an improvement at 6 months which continued at 12 months also (42). Similarly, a case report from 2018 of a 24-year-old male with UC and an anterior uveitis flare was started on vedolizumab (74). At his 1-year follow up, he had not developed another uveitis flare and a repeat colonoscopy demonstrated normal mucosa (74).

However, a retrospective study on patients with IBD comparing EIMs in patients receiving vedolizumab versus anti-TNF therapies demonstrated that patients with CD on vedolizumab were 28% more likely to develop any EIMs. The incidence rate ratio of episcleritis/scleritis was 2.51 (95% CI, 1.02-6.14) and uveitis was 2.89 (95% CI, 1.35-6.18). Patients with UC were not more likely to develop any type of EIMs, but were more likely to develop other, non-ophthalmic EIMs including aphthous stomatitis, pyoderma gangrenosum, and PSC (89). This indicates that vedolizumab may have limited efficacy outside of the gut and may explain why it is not commonly used (89).

### 5.4 Cytokine inhibitors

IL-23 therapies have been explored to treat IBD and uveitis since IL-23 was found to be elevated in IBD and NIU (32). Ustekinumab (Stelara), which is an anti-interleukin-23, has been used to treat patients with uveitis and CD. Two female patients with ileocolonic CD who had undergone ileocecal resection and had multiple flares of severe bilateral non-infectious uveitis yearly despite adalimumab treatment were treated with an induction dose of ustekinumab and maintenance treatment every 8 weeks for at least 6 months without recurrence of NIU. Ustekinumab may, thus, be an effective treatment for patients with severe recurrent NIU and for patients in whom anti-TNFs are either contra-indicated or ineffective (55).

Similarly, in an international multicenter retrospective study of IBD patients who were treated with either ustekinumab or vedolizumab, one patient developed episcleritis while on vedolizumab while no patients developed new inflammatory eye disease while on ustekinumab after 1 year of treatment (13). Additionally, more patients developed arthralgias while on vedolizumab, and 2 patients on vedolizumab stopped treatment due to arthralgias while no patients on ustekinumab discontinued treatment (13).

These cytokine therapies have thus far demonstrated superiority to the prior medications; however, long term data in larger populations remains to be gathered.

### 5.5 JAK inhibitors

JAK inhibitors are relatively new medications in the treatment of EIMs of IBD which are being explored due to the JAK pathway’s involvement in signal transduction for multiple cytokines in the setting of inflammation (25). The OCTAVE trial, evaluating tofacitinib, demonstrated either improvement of active disease from all EIMs or stability from baseline after 52 weeks of treatment (25). Based on this trial, the JAK inhibitors present a promising future for the treatment not only of uveitis, but for many EIMs.

### 5.6 Exosomes

The newest technologies that seek to treat EIMs in IBD are mesenchymal stem cell-derived exosomes (MSC-EXOs). While these treatments are still in preclinical trials, they demonstrate promising outcomes. Specifically, MSC-EXOs suppress the inflammatory response by decreasing CD3+ T cells in the retina and by preventing the migration of macrophages to the retina of mice (90). The studies also demonstrated a reduction in the inflammation of IBD and uveitis in mice when targeting Th1 and Th17 cells thus showing the importance of these cells for EIMs and IBD, and demonstrating a potential drug target (90). As these treatments progress in research, it will be important to ensure the accessibility of these medications to the patients for whom they were made.

### 5.7 Rifabutin

Historically, rifabutin was used as a treatment for uveitis due to a theory (47, 49) suggesting that *Mycobacterium paratuberculosis* contributed to the pathogenesis of uveitis. However, there have been cases (62, 65) of patients developing ocular inflammation when on rifabutin and clarithromycin. This disease process has been coined “rifabutin-associated uveitis.” Worsening ocular inflammation arises leading to a hypopyon which is unusual in uveitis alone (62, 65). While the pathogenesis of rifabutin-associated uveitis is unknown, physicians should avoid using rifabutin in patients with IBD-associated uveitis for risk of worsening the ocular inflammation especially when other immunomodulators can be used instead. However, if a patient presents with rifabutin-associated uveitis, then the authors of the case reports suggest resolution of symptoms with systemic steroids (62, 65).

### 5.8 Colectomy

Though some have argued that recurrence of uveitis symptoms after colectomy is unlikely (54), there is a case report of patient who developed granulomatous uveitis on post-colectomy day 1 (58). This case also demonstrated the importance of rapid recognition and treatment of granulomatous uveitis to avoid enucleation (57). Similarly, another case report indicated that ocular inflammation after colectomy may be due to recurrent bowel inflammation which should be evaluated (59). A retrospective study found that there was no significant difference in ocular manifestations after undergoing or not undergoing a colectomy (82). Given the lack of definitive data, it is not clear that a colectomy should be performed to eliminate uveitic disease. Furthermore, uveitis should not be ruled out as a cause of ocular symptoms after a colectomy.

## 6 Conclusion

Inflammatory Bowel Disease and its O-EIMs can be a debilitating disease for patients. It is, unfortunately, difficult to identify a specific chronicity in terms of the timing of ocular inflammation and gut disease, so it remains important for physicians to efficiently evaluate patients who may be at risk for developing O-EIMs secondary to IBD utilizing a comprehensive review of symptoms. We found that adults and children with CD were more likely to present with O-EIMs as opposed to patients who had UC. Additionally, women rather than men with IBD were more likely to present with O-EIMs as is common with other autoimmune conditions. Patients with arthritis and skin EIMs are more likely to develop ocular manifestations. Education is needed to increase the awareness of vision threatening diseases secondary to IBD to primary care providers and gastroenterologists. Nonetheless, patients with IBD do not need to be routinely screened by an ophthalmologist for ocular manifestations if there are no ophthalmic complaints. However, if a patient presents with 2 of the 3 symptoms (i.e., ophthalmic complaints, skin or joint complaints, and gastrointestinal complaints), the physician should have a lower threshold for referral to gastroenterology for evaluation.

In terms of specific ocular presentations, episcleritis and anterior uveitis are the most common O-EIMs. However, most patients present with non-specific eye complaints, that should be evaluated and referred as appropriate.

There are many effective medications for the treatment of O-EIMs in patients with IBD. Non-steroidal anti-inflammatory drugs are relatively contraindicated in patients with inflammatory bowel disease due to the increased risk of erosion of the lining of the bowel. Corticosteroids are a reasonable short-term therapy until corticosteroid-sparing therapy can be initiated as a better long-term treatment option. Anti-metabolites and anti-TNF agents have demonstrated equal efficacy, though anti-metabolites may cause more side-effects. Of the newer therapies, cytokines inhibitors and JAK inhibitors have demonstrated efficacy against IBD and uveitis, and more data is needed for exosome therapies.

Patients with IBD can present with a varying amount of ocular inflammation that may be independent of bowel disease activity. It is important for patients with IBD to have optimized interdisciplinary care to determine the best course of treatment for manifestations of inflammation in multiple organs. More research needs to be done to assess the relationship between IBD and O-EIMs in order to streamline a diagnosis and treatment in patients who suffer from these debilitating conditions.

The strengths of this article are the extended time period of literature that was reviewed as well as the inclusion of information from multiple populations and across multiple subspecialties. The limitations of this paper included limited articles on the prevalence and incidence on uveitis in IBD patients.

## Author contributions

MRD: Investigation, Writing – original draft, Writing – review & editing. GADO’K: Supervision, Writing – review & editing.
